# Extreme inbreeding in a European ancestry sample from the contemporary UK population

**DOI:** 10.1038/s41467-019-11724-6

**Published:** 2019-09-03

**Authors:** Loic Yengo, Naomi R. Wray, Peter M. Visscher

**Affiliations:** 1 Institute for Molecular Bioscience, The University of Queensland, QLD 4072 Brisbane, Australia; 2 Queensland Brain Institute, The University of Queensland, Brisbane, 4072 Australia

**Keywords:** Inbreeding, Consanguinity, Behavioural genetics, Population genetics

## Abstract

In most human societies, there are taboos and laws banning mating between first- and second-degree relatives, but actual prevalence and effects on health and fitness are poorly quantified. Here, we leverage a large observational study of ~450,000 participants of European ancestry from the UK Biobank (UKB) to quantify extreme inbreeding (EI) and its consequences. We use genotyped SNPs to detect large runs of homozygosity (ROH) and call EI when >10% of an individual’s genome comprise ROHs. We estimate a prevalence of EI of ~0.03%, i.e., ~1/3652. EI cases have phenotypic means between 0.3 and 0.7 standard deviation below the population mean for 7 traits, including stature and cognitive ability, consistent with inbreeding depression estimated from individuals with low levels of inbreeding. Our study provides DNA-based quantification of the prevalence of EI in a European ancestry sample from the UK and measures its effects on health and fitness traits.

## Abstract (web summary)

Mating between first or second-degree relatives is prohibited in most countries, yet it occurs and is under-studied. Here, Yengo et al. use large runs of homozygosity from the UK Biobank resource to provide DNA-based quantification of extreme inbreeding and its consequence for health and other complex traits.

## Introduction

Mating between close relatives, that is inbreeding, is reported in many species to yield deleterious outcomes, such as reduced fertility^1–4^, stature^2,4–10^ and lifespan^2^. In humans, consanguineous mating leads to higher childhood mortality^3,11,12^ and to adverse effects on traits such as lung function^4,10,13^ and cognitive ability^4,8,10,13^. Because of its detrimental consequences, also referred to as inbreeding depression, a number of species have developed inbreeding avoidance mechanisms to limit its effects^14^. In humans, inbreeding avoidance mechanisms, include cultural and religious taboos on incest, and laws explicitly forbidding certain types of mating. For instance, the Sexual Offences Act (2003) in the UK specifically forbids mating between first-degree (parent–offspring or fullsibs (FS), i.e., coefficient of relationship of 0.5) and second-degree (halfsibs (HS), grandparent–grandchild, avuncular or double-first cousins, i.e., coefficient of relationship of 0.25) relatives; and also forbids mating between step and relatives when one of the family members is below 18 years old. Cultural, legal, religious and health-related constraints strongly weigh on the ability to observe, and therefore study the causes and consequences of inbreeding between first- and second-degree relatives, hereafter referred to as extreme inbreeding (EI). A number of previous studies have attempted to quantify the prevalence and incidence of EI^15–20^. However, as underlined by van den Berghe^21^, the estimates which they produced are questionable given the “disinclination of family members to report incest when it occurs, and the countervailing bias of many scholars and crusaders to magnify the problem on which they build their career”. Add to these limitations, the relatively small size of these studies (often <1000 participants) and the discrepancies between them with respect to the definition of EI, as some of these studies included mating between step-relatives^21^. Here, we leverage a large observational study of ~450,000 participants, the UK Biobank (UKB), to quantify EI and its consequences in contemporary European descents from the UK population. We compare our estimates with the prevalence of police-recorded cases of incest offences reported in the Crime Survey for England and Wales (CSEW) between April 2002 and March 2017. We also characterise the distribution of runs of homozygosity (ROHs) in EI cases and assess its consistency with theoretical predictions. Finally, we characterise the phenotypic consequences of EI on a number of health-related traits measured in UKB participants.

## Results

### Prevalence of EI in European descents from the UKB

We previously identified^22^ 456,426 individuals of European ancestry among the 487,409 UKB participants who have been genotyped. Ancestry was called in our previous study using projected principal components analysis based on known ancestry and whole-genome sequence data from 2504 participants of the 1000 Genomes Project^23^ (Methods). Given that 12 participants had retracted consent, we only analysed 456,414 UKB participants in the present study. We used 301,412 quality-controlled genotyped single-nucleotide polymorphisms (SNPs) to call ROHs using the PLINK software (Methods). As in previous studies^4,8,10^, ROHs were defined as homozygous >1.5 Mb long genomic segments (Methods). We then estimated for each study participant the percentage of their autosome comprising ROHs as a measure of inbreeding. Such inbreeding measure, hereafter denoted *F*_ROH_, is a well-established predictor of pedigree inbreeding^24,25^. Following guidelines from the American College of Medical Genetics and Genomics (ACMG)^26,27^, EI was called for individuals with *F*_ROH_ > 0.1. The use of both *F*_ROH_ as a measure of inbreeding and of this threshold are recommended by the ACMG for detecting suspected consanguinity between parents.

We thus identified 125 unrelated participants (65 males and 60 females) whose genomes are consistent with their parents being first- or second-degree relatives. That represents a prevalence of EI ~0.03%, i.e., ~1/3652 (95% confidence interval—CI_95%_: [1/4428–1/3106]). As a sensitivity analysis, and consistent with theory predicting much longer ROHs under EI, we re-estimated the prevalence of EI considering only ROHs > 2 Mb or >5 Mb long. Using these alternative definitions of ROH, also recommended in the ACMG guidelines, we detected 115 (prevalence of ~1/3969; CI_95%_: [1/4857–1/3355]) and 98 (prevalence of ~1/4658; CI_95%_: [1/5807–1/3887]) cases of EI, respectively. We also estimated the prevalence of EI using allele-frequency based inbreeding measures or using ROHs detected on both autosome and X-chromosomes of female participants. (Supplementary Table 1). Given that the latter estimates of the prevalence of EI are not statistically distinct (paired *t* test: *p* > 0.05) from our first estimate based on ROHs > 1.5 Mb, we will hereafter only consider ROHs > 1.5 Mb.

We then compared our estimate of the prevalence of EI with the prevalence of incest offences reported in the CSEW between April 2002 and March 2017. That survey reports a total of 11,196 cases of police-recorded incest offences over this time period (URLs). Relative to the population of England and Wales, which varied from 52,602,200 to 58,744,600 between those years (URLs), this represents a prevalence ranging from ~1/5247 (CI_95%_: [1/5346–1/5151]) to 1/4699 (CI_95%_: [1/4787–1/4612]). The latter estimate is of the same order of magnitude as our estimated prevalence of EI in the UKB although these two estimates are based on different time periods (births 1938–1967 in the UKB vs. reports 2002–2017 in the CSEW). We then compared the mean years of birth among EI cases with the rest of UKB participants and found no statistical difference (*p* = 0.11). That suggests that the prevalence of EI is relatively unchanged over time although mean inbreeding coefficients have significantly decreased over the years (correlation between year of birth and *F*_ROH_: *r* = −0.01%; Pearson’s correlation test *p* = 5.5 × 10^−14^). However, it is important to note that the prevalence of EI and that of police-recorded incest offences cannot naïvely nor strictly be compared because (i) only an unknown but likely small fraction of incest cases are reported to the police, (ii) not all cases of incest would result in viable offspring as observed in this study, and (iii) viable offspring with severe cognitive impairment due to inbreeding are unlikely to enrol themselves as participants in the UKB.

Fry et al.^28^ previously reported that the UKB is not representative of the entire UK population, as it notably, includes healthier and more educated participants than the average population. Such an ascertainment on traits which are negatively correlated with inbreeding (e.g., educational attainment (EA) or height^28^), may lead the prevalence of EI in the UKB to be an underestimation of the actual prevalence of EI in the UK population. As a consequence, our estimate of prevalence of EI is likely conservative, although the magnitude of the underestimation is difficult to predict as it depends on many other unknown factors which might differ between UKB participants and the general population.

### Deconvolution of underlying mating types

**Fig. 1:**
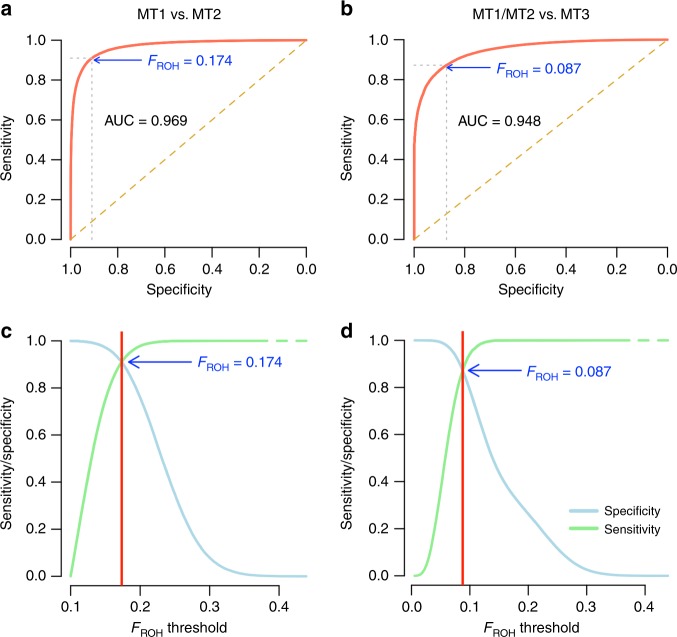
Predictive performances of FROH to discriminate different types of inbreeding: mating type 1 (MT1: parent-offspring or fullsibs mating), mating type 2 (MT2: halfsibs, avuncular, grandparent-grandchild or double-first cousins mating) or mating type 3 (MT3: first-cousins mating). Panels **a** and **c** correspond to the comparison of MT1 and MT2, while panels **b** and **d** correspond to the comparison of MT1 and MT2 on the one hand and MT3 on the other hand. Predictive statistics assessed are the area under the receiver characteristics operating curve (AUC), the sensitivity to detect MT1 over MT2 (true positive rate) and specificity to distinguish MT1 from MT2 (true negative rate). FROH>0.17 yields a sensitivity and specificity >0.92 to discriminate MT1 from MT2; and FROH>0.087 yields a sensitivity of ~0.94 and a specificity of ~0.79 to discriminate MT1 or MT2 from MT3

We next estimated the proportion of EI cases born from mating between first-degree relatives (mating type 1; MT1) vs. second-degree relatives (MT2) using a threshold-based approach based on *F*_ROH_. To determine an optimal threshold, we simulated inbreeding under MT1 and MT2 using phased genotypes from 972 unrelated UKB participants (Methods, Supplementary Table 2). These 972 UKB participants are the offspring from 972 independent parent–offspring (PO) trios identified in the UKB^29^. Over ~20,000 simulation replicates (one replicate is one simulated EI case) we found that *F*_ROH_ as a predictor of underlying mating type (MT1 vs. MT2) yields an area under the receiver operating characteristic curve (AUC) of ~0.97 and that using *F*_ROH_ > 0.17 as a threshold yields optimal sensitivity and specificity both >0.92 (Fig. 1). Using this threshold, we therefore identified 54/125 (i.e., ~43.2%) EI UKB cases whose parents are most likely first-degree relatives. It is worth noting that complex inbreeding loops between second degree-relatives may also lead to extreme values of *F*_ROH_. However, mating between first-degree relatives remains a more parsimonious explanation of the empirical observations, in particular in a population of European ancestry where such complex inbreeding loops are uncommon.

We further attempted to quantify the proportion of MT1 born from PO vs. FS mating (*π*_*PO*/*FS*_). Given that the theoretical expectation of *F*_ROH_ is 0.25 both under PO and FS mating, we found, as expected, that *F*_ROH_ alone cannot discriminate PO from FS in our simulations (AUC of ~0.5).

However, *F*_ROH_ being proportional to the cumulative length of ROHs across the genome also implies that the same value of *F*_ROH_ could reflect either fewer larger ROHs, or more smaller ROHs. Therefore, we investigated if the numbers of ROHs detected (*N*_ROH_) under PO or FS mating are different and if so can discriminate those two types of mating. We found on average over ~20,000 simulation replicates that *N*_ROH_ ~45 ROHs are detected in offspring of FS mating as compared to *N*_ROH_ ~38 ROHs detected in offspring of PO mating (Table 1). Moreover, ROHs detected in offspring of PO mating were on average ~2.7 Mb longer than ROHs detected under FS mating (Table 1). Consistent with these observations, we found that *N*_ROH_ as a predictor of mating type yields a discriminative AUC of ~0.81, with the optimal threshold of >41 yielding a sensitivity of ~0.77 and specificity of ~0.69. Using that threshold we predict that 24/54 (i.e., *π*_*PO*/*FS*_ ~44.4%; CI_95%_: [31.2–57.7%]) EI cases with *F*_ROH_ > 0.17 are likely offspring of parent–offspring mating. We also considered an alternative approach that aims at directly estimating the proportion of EI cases born from PO vs. FS mating from modelling the length distribution of ROHs (Methods). We applied this method to 2244 ROHs segments detected in 54 EI cases with *F*_ROH_ > 0.17 and estimated that *π*_*PO*/*FS*_ ~67.6% (CI_95%_: [45.2–90.1%]). To confirm this finding, we analysed the distribution of *F*_ROH_ from X-chromosome ROHs (hereafter denoted *F*_ROH-X_) in 26 female EI cases with *F*_ROH_ > 0.17. This analysis is justified by the fact that the theoretical expectation of *F*_ROH-X_ equals 0.5 under PO mating vs. 0.25 under FS mating. We first stratified these 26 female EI cases into two groups (Group 1 and Group 2) depending on whether the likelihood of their autosomal segments lengths is larger under PO mating or under FS mating. More specifically, Group 1 (*N* = 10) and Group 2 (*N* = 16) contain female EI cases predicted to be offspring of FS and PO, respectively (Supplementary Fig. 1) from the length distribution of autosomal ROHs. The mean *F*_ROH-X_ in Group 2 is 0.53 (CI_95%_: [0.41–0.65]), consistent with PO mating, while the mean *F*_ROH-X_ in Group 1 is 0.34 (CI_95%_: [0.19–0.49]), which is consistent with FS mating, although standard errors are large. Altogether, we found that between 44.4 and 67.6% of EI cases with *F*_ROH_ > 0.17 are likely offspring of PO mating.

**Table 1:** Mean number and length of runs of homozygosity (ROHs) detected in participants from the UK Biobank (UKB), including extreme inbreeding (EI) cases (defined as *F*_ROH_ > 0.1) and unrelated EI controls (defined as *F*_ROH_ < 0.01). We also report the mean and length of ROHs in simulated data under various mating types

	Sample size	Mean number of ROHs per individual (SD)	Mean length of ROHs in Mb (SD)	Mean *F*_ROH_ (SD)
Observed ROHs in UKB participants^a^				
EI cases (*F*_ROH_ > 0.1)	125	33.6 (10.3)	14.8 (15.6)	0.172 (.07)
Unrelated EI controls (*F*_ROH_ < 0.01)	345,276	4.9 (2.2)	2.1 (0.8)	0.003 (.002)
ROHs from simulated data under various mating types				
Parent–offspring mating (PO)	19,062	38.1 (5.9)	17.6 (18.5)	0.253 (.054)
Fullsibs mating (FS)	19,065	45.2 (5.9)	14.9 (15.4)	0.254 (.046)
Halfsibs mating (HS)	19,048	25.0 (5.5)	13.6 (15.1)	0.128 (.040)
Uncle/aunt–niece/nephew mating (AV)	19,108	28.3 (5.6)	12.0 (13.0)	0.127 (.036)
Grandparent–grandchild mating (GP)	19,025	24.9 (5.4)	13.6 (15.1)	0.128 (.039)
Double first cousins mating (DC)	19,080	31.6 (5.7)	10.8 (11.3)	0.128 (.032)
First cousins mating (FC)	19,061	18.1 (4.7)	9.6 (10.9)	0.065 (.025)
Mating between unrelated parents (UN)	18,912	4.8 (2.1)	2.0 (0.7)	0.004 (.002)
Mating type 1 (MT1: PO or FS)	38,127	41.7 (6.9)	16.1 (16.9)	0.254 (.050)
Mating type 2 (MT2: HS or AV or GP or DC)	76,261	27.4 (6.1)	12.4 (13.6)	0.128 (.037)
Mixture of MT1 and MT2 mating (Mixture proportion: 54/125)	139,310	33.6 (9.6)	14.0 (15.2)	0.182 (.075)

*SD* standard deviation

^a^Mean differences between EI cases and controls are statistically significant at *p* < 10^−10^

We simulated inbreeding between first-cousins (hereafter denoted MT3) in order to quantify the ability of the *F*_ROH_ > 0.1 threshold recommended by the ACMG guidelines to discriminate MT1 or MT2 from MT3. We recall here that the coefficient of relationship between first-cousins is 0.125, and therefore the expected inbreeding coefficient of their offspring is E[*F*_ROH_] = 0.5 × 0.125 = 0.0625. Also, MT3 is legal in most countries and thus more common in the population. We found over ~20,000 simulation replicates that *F*_ROH_ yields an AUC of ~0.95, and that using *F*_ROH_ > 0.1 as a threshold yields a sensitivity of ~0.94 and a specificity of ~0.79 to discriminate MT1 or MT2 from MT3 (Fig. 1). This, therefore, suggests that ~8/125 EI cases identified (i.e., ~6.4%) in this study could in fact be offspring of first-cousins mating. Hill and Weir^30^ derived that the theoretical standard deviation of inbreeding coeffcients of offspring of first-cousins is ~0.024. Therefore, assuming under MT3 that *F*_ROH_ is normally distributed with mean 0.0625 and standard deviation 0.024, follows that the probability of *F*_ROH_ > 0.1 equals ~5.9%, which is consistent with our simulations.

### Distribution of ROH in EI cases

**Fig. 2:**
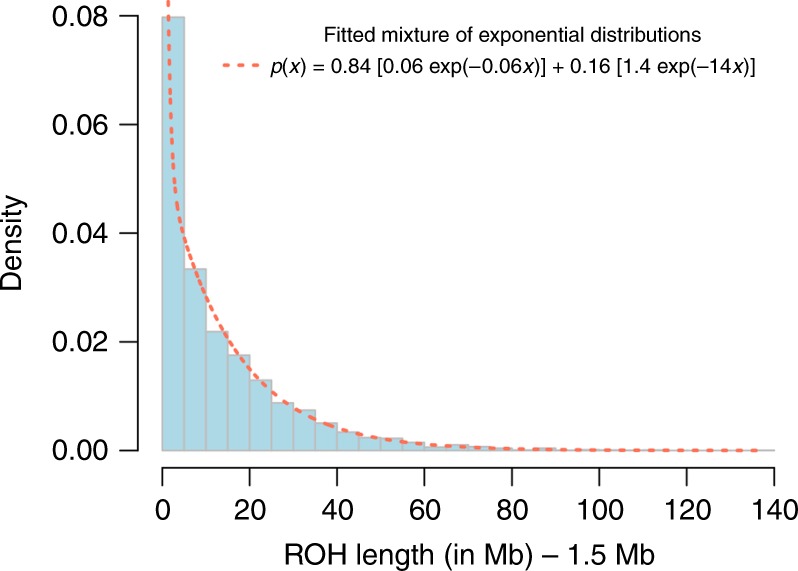
Histogram of the lengths of 4,196 runs of homozygosity (ROHs) detected in 125 EI cases (*F*_ROH_ > 0.1). Each length was subtracted 1.5 Mb (i.e., minimum length used to detect ROHs) before mixture distribution was fitted. A 84:16 mixture of two exponential distributions with means ~15.7 Mb (rate = 1/15.7 ~0.06) and ~0.72 Mb (rate = 1/0.72 ~1.4), respectively was found to best fit the observed length distribution (dotted line)

**Fig. 3:**
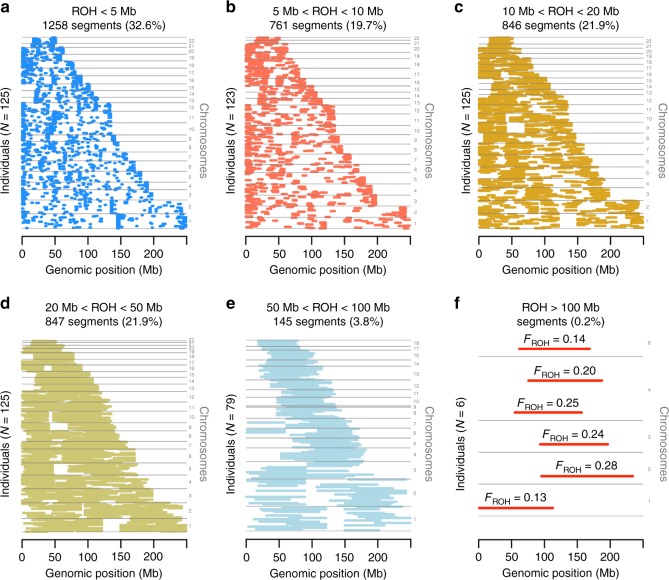
Chromosomal and positional distribution of runs of homozygosity (ROHs) detected in 125 EI cases (*F*_ROH_ > 0.1). Each row, with possibly multiple segments, represents a unique participant. Segments are groups by autosomal chromosomes from chromosome 1 (bottom of each panel) to chromosome 22 (top of each panel) ROHs are grouped in 6 length categories: between 1.5 and 5 Mb (**a**), between 5 and 10 Mb (**b**), between 10 and 20 Mb (**c**), between 20 and 50 Mb (**d**), between 50 and 100 Mb (**e**), and above 100 Mb (**f**). **f** also show inbreeding coefficients of individuals harbouring the largest ROHs

As expected, we found that EI cases harboured significantly more and significantly longer ROHs than EI controls (*F*_ROH_ < 0.01) in the population (Table 1). On average, we detected *N*_ROH_ ~33.6 ROHs in EI cases vs. ~4.9 ROHs in EI controls. The mean length of ROHs was *L*_ROH_ ~14.8 Mb in EI cases vs. ~2.1 Mb in EI controls. Both mean numbers and mean lengths of ROHs detected are consistent with our simulations of EI (mean *N*_ROH_ ~33.6 and *L*_ROH_ ~14.0; Table 1). We represent in Fig. 2 the histogram of ROHs length in EI cases, and report in Fig. 3, a few examples of very large ROHs (>100 Mb) covering ~50% of an entire chromosome. We also report X-chromosome ROHs detected 54/125 female EI cases in Supplementary Fig. 2.

Previous theoretical studies have often considered the length of genomic segments homozygous by descent (HBD) to follow an exponential distribution^31,32^. These studies generally relied on specific assumptions regarding recombination map functions, like Haldane or Kosambi map functions, which yield tractable algebraical simplifications. However, empirical evidence supporting these assumptions remains limited. Moreover, some of these simplifying assumptions like that of independence between the lengths and the numbers of HBD segments have also been criticised^33^. Here, we used an empirical approach to estimate the length distribution of ROHs segments detected in EI cases using a mixture of exponential distributions. Given that only ROHs larger than 1.5 Mb were detected, we modelled the distribution of lengths minus 1.5 Mb and not directly the length distribution, which would better fit a mixture of truncated exponential distributions (Methods). Mixtures of exponential distributions represent a flexible family of probability distributions, from which the exponential distribution is a special case. We selected the number of mixture components best fitting the data using the Bayesian Information Criterion (BIC). To calibrate our inference, we first estimated the length distribution of >282,635 simulated true HBD segments under various mating types. Our simulations are based on observed recombination maps from the 1000 Genomes Project^23^, and therefore do not make additional assumptions regarding recombination rates (Methods). We found for all simulated mating types that BIC selects two mixture components, which suggests that the single exponential distribution is likely too simple to characterise the length distribution of HBD segments. Of note, mixtures of two exponential distributions also yield a better fit than gamma distributions that have previously also been proposed^1^. Similarly, we estimated the length distribution of >99,794 ROHs detected in our simulated data. We found consistently that the length distribution of simulated ROHs is also well characterised by a mixture of two exponential distributions. We report in Table 2, the parameters of the mixture distributions estimated from true HBD segments and from ROHs. We then estimated the length distribution of the 4196 ROHs detected over all EI cases. We found this distribution to fit a 84:16 mixture of exponential distributions with means ~15.7 Mb (larger component) and ~0.7 Mb (smaller component), respectively (Table 2; Fig. 2). Overall, our findings suggest that the length distribution of HBD segments and ROHs can be well approximated with a mixture of two exponential distributions.

**Table 2:** Parameters of mixtures of exponential distributions estimated from observed length distributions of homozygous-by-descent (HBD) genomic segments and runs of homozygosity (ROH)

Simulated true HBD segments	Mating type	Number of segments (individuals)	Mean (SD) number of segments per individual	*π*_1_	*π*_2_	*λ*_1_	*λ*_2_	1/*λ*_1_	1/*λ*_2_
	PO	620,017 (19,062)	32.5 (5.1)	0.75	0.25	0.038	0.165	26.6	6.1
	FS	791,483 (19,065)	41.6 (5.4)	0.71	0.29	0.047	0.186	21.4	5.4
	HS	395,500 (19,096)	20.8 (4.7)	0.71	0.29	0.047	0.182	21.5	5.5
	AV	481,752 (19,129)	25.2 (5.1)	0.69	0.31	0.056	0.208	17.9	4.8
	GP	393,584 (19,081)	20.8 (4.8)	0.71	0.29	0.047	0.186	21.3	5.4
	DC	566,552 (19,082)	29.8 (5.3)	0.65	0.35	0.064	0.225	15.6	4.4
	FC	282,635 (19,430)	14.9 (4.3)	0.65	0.35	0.064	0.224	15.5	4.5
Simulated ROHs	PO	726,247 (19,062)	38.1 (5.9)	0.88	0.12	0.055	1.309	18.3	0.8
	FS	862440 (19,065)	45.2 (5.9)	0.87	0.13	0.066	1.015	15.2	1.0
	HS	476,464 (19,048)	25.0 (5.5)	0.81	0.19	0.068	1.797	14.7	0.6
	AV	540,866 (19,108)	28.3 (5.6)	0.82	0.18	0.079	1.656	12.6	0.6
	GP	474,085 (19,025)	24.9 (5.4)	0.81	0.19	0.068	1.802	14.7	0.6
	DC	602,297 (19,080)	31.6 (5.7)	0.83	0.17	0.091	1.530	11.0	0.7
	FC	345,251 (19,061)	18.1 (4.7)	0.73	0.27	0.093	1.946	10.8	0.5
	UN	90,794 (18,912)	4.8 (2.1)	0.72	0.28	2.895	0.984	0.30	1.0
ROHs detected in EI cases	Mixture	4196 (125)	38.9 (12.9)	0.84	0.16	0.064	1.385	15.7	0.7

Estimated parameters, include mixture proportions (*π*_1_ and *π*_2_ = 1 − *π*_1_) and rates (*λ*_1_ and *λ*_2_ in Mb^−1^) of the two exponential distributions composing the mixture. Mixture of exponential distributions were fitted to the length distribution of simulated true HBD segments, of simulated ROHs and of ROH detected in 125 cases of extreme inbreeding (EI). Simulations were performed under various mating types: parent–offspring mating (PO), full-sib mating (FS), half-sib mating (HS), avuncular mating (AV), grandparent–grandchild mating (GP), double first cousins (DC), first-cousins mating (FC) and mating between unrelated individuals (UN). *SD* standard deviation

Another observation in our simulations was that that the mean number of ROHs detected in an individual was larger than the number of true HBD segments simulated. This somewhat counterintuitive observation is explained by the fact that HBD were defined as segments identical-by-descent (from parents to offspring), while ROHs were re-estimated from the genotypes of simulated offspring. As a consequence, although simulated offspring of matings between unrelated parents have exactly zero HBD segments, they still harbour ROHs > 1.5 Mb given that their chromosomes were sampled from 972 existing UKB participants. Despite not being closely related (genomic relationship (GRM) < 0.05), these 972 UKB participants are still likely to have a distant common ancestor (>25 generations ago), which would lead to detection of ROHs > 1.5 Mb in their (simulated) offspring. We found that simulated offspring of matings between unrelated parents had on average 4.8 ROHs > 1.5 Mb (Table 1). If we subtract that number (i.e., 4.8 ROHs) from the mean number of ROHs detected under simulated inbred matings (Tables 1 and 2), we now find very consistent mean numbers of ROHs and HBD segments per individual. More specifically, for each simulated inbred mating we find, after this correction, 32.5 HBD vs. 33.3 ROH for PO mating, 41.6 HBD vs. 40.4 ROH for fullsibs mating, 20.8 HBD vs. 20.2 ROH for HSs mating, 25.2 HBD vs. 23.5 ROH for avuncular mating, 20.8 vs. 20.1 ROH for grandparent–grandchild mating, 29.8 vs. 26.8 ROH for double-first cousin mating and 14.9 HBD vs. 13.3 ROH for first-cousin mating.

### Phenotypic consequences of EI

We quantified the consequences of EI on multiple traits measured in the UKB. We first analysed ten control traits with prior evidence of inbreeding depression^4,8,10,13^. Those ten traits are height, hip-to-waist ratio (HWR), handgrip strength (HGS; average of left and right hand), lung function measured as the peak expiratory flow (PEF), visual acuity (VA), auditory acuity (AA), number of years of education (EA), fluid intelligence score (FIS), cognitive function measured as the mean time to correctly identify matches (MTCIM) and fertility measured as the number of children (NCh). We performed linear regressions of these traits on the EI status adjusted for age at recruitment, recruitment centre (treated as a categorical factor), sex, year of birth (treated as a continuous variable), genotyping batch (treated as a factor), socioeconomic status measured by the Townsend deprivation index and population structure measured by ten genetic principal components estimated from HM3 SNPs. As expected, we found that EI cases had a reduced mean in these ten traits as compared to EI controls. More specifically, we found phenotypic means in EI cases to be between 0.3 and 0.7 standard deviation below the population mean (Table 3). Note, that under normality assumptions, between ~25 and ~40% of the population has a phenotype below 0.7 and 0.3 standard deviations below the mean, respectively. Despite the small sample size of 125 EI cases, the reduction was statistically significant (Wald-test *p* < 0.5/10 = 0.005) for 7 out the 10 traits (Table 3). We also specifically estimated the inbreeding load (often denoted *B*), which represents the number of loci with deleterious alleles that would cause one death on average if made homozygous^3^. As previously recommended^34^, we estimated *B* using Poisson regression of the number of children engendered onto *F*_ROH_. Poisson regression was performed using a logarithmic link function as also previously recommended^34^ and adjusted for the same covariates listed above. For this analysis, we used the entire distribution of *F*_ROH_, (i.e., includes both EI cases and EI controls) and found an estimate of *B* ~1.46 (CI_95%_: [0.87–2.05]; Wald-test *p* = 1.3 × 10^−6^; Table 3). The effect of inbreeding on fertility of the resulting inbred offspring, that we have quantified here, has been previously detected in humans^35^. However, the latter study did not provide an estimate of inbreeding load that can be directly compared with ours. Nonetheless, we found that our estimate is consistent with estimates of inbreeding load on survival of offspring from inbred mating in humans^3,36^ and other species^34,37^, although these are different traits.

**Table 3:** Association between extreme inbreeding (EI) and multiple traits measured in UK Biobank participants (125 EI cases vs. 345,276 EI controls)

Traits (unit: trait SD)	Mean in EI cases	Mean in controls	Effect size (unit: trait SD)	Extrapolated effect size (unit: trait SD for 100% inbreeding)	Standard error (s.e.)	*p* Value
PEF	−0.651	0.005	−0.656	−3.88	0.099	2.8 × 10^−11^
Height	−0.404	0.012	−0.417	−2.46	0.090	3.2 × 10^−6^
HGS	−0.395	0.004	−0.441	−2.35	0.091	1.2 × 10^−5^
FIS	−0.570	0.010	−0.581	−3.43	0.152	1.4 × 10^−4^
MTCIM	−0.334	0.003	−0.337	−1.99	0.091	2.0 × 10^−4^
AA	−0.557	0.002	−0.559	−3.31	0.164	6.7 × 10^−4^
EA	−0.260	0.023	−0.283	−1.67	0.089	1.5 × 10^−3^
VA	0.370	0.003	−0.373	−2.21	0.179	0.037
NCh	−0.230	−0.009	−0.221	−1.31	0.089	0.013
HWR	−0.640	0.005	−0.170	−1.01	0.090	0.058
Polygenic predictor of EA*	−0.259	−0.262	4.9 × 10^−4^	N/A	0.018	0.978
			RR [log(RR)]		s.e. of log(RR)	P-value
NCh	1.54	1.78	0.23 [−1.46]	N/A	0.302	1.3 × 10^−6^
NDIS	12.2	6.90	1.44 [0.36]	N/A	0.089	3.6 × 10^−5^
NDIS*	13.9	8.90	1.34 [0.29]	N/A	0.083	4.4 × 10^−4^
NDIS parents	2.16	2.24	0.96 [−0.04]	N/A	0.065	0.507

Mean *F*_ROH_ is ~0.172 (SD: 0.067) in EI cases and ~0.003 (SD: 0.002) in EI controls. Effect sizes were estimated using either linear regression or overdispersed Poisson regression (latter for the following traits NCh, NDIS, NDIS* and NDIS parents) of the trait on the EI binary status. Extrapolated effect sizes in trait SD for 100% inbreeding were obtained by dividing estimated effect size by the difference in mean *F*_ROH_ between EI cases and EI controls, i.e., ~0.17. Traits analysed, include peak expiratory flow (PEF), standing height, handgrip strength (HGS), fluid intelligence score (FIS), mean time to correctly identify matches (MTCIM), auditory acuity (AA), number of years of education or educational attainment (EA), visual acuity (VA), number of children (NCh), hip-to-waist ratio (HWR), number of diseases diagnosed (NDIS) based on the International Classification of Diseases, Tenth Revision (ICD10). NDIS* refers to NDIS in individuals with at least one disease diagnosed. We also compared the number of disease-groups UKB participants reported their parents to be affected with (NDIS parents). *RR* relative risk, *SD* standard deviation. Estimates were adjusted for age at recruitment, recruitment centre (treated as a categorical factor), sex, year of birth (treated as a continuous variable), genotyping batch (treated as a factor), socioeconomic status measured by the Townsend deprivation index and population structure measured by ten genetic principal components estimated from HM3 SNPs. Polygenic predictor of EA was calculated using summary statistics from the Lee et al. (2018) study (excluding the UKB) and also adjusted for ten principal components. Inbreeding load for NCh was estimated as *B* = −log(RR) = 1.46 (95% confidence interval: [0.87–2.05]).

**Fig. 4:**
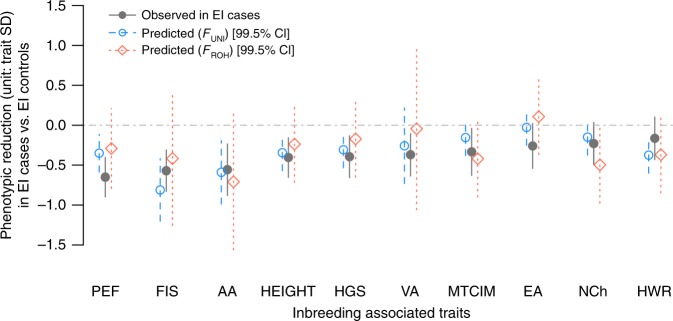
Phenotypic reduction (in trait standard deviation; SD) observed in 125 extreme inbreeding (EI: *F*_ROH_ > 0.1) cases compared to 345,276 unrelated EI controls (*F*_ROH_ < 0.01). Observed means for EI cases and controls are reported in Table 3. Phenotypic reduction was assessed for ten traits: auditory acuity (AA), fluid intelligence score (FIS), peak expiratory volume (PEF), hip-to-waist ratio (HWR), visual acuity (VA), height, cognitive ability measured as the mean time to correctly identify matches (MTCIM), handgrip strength (HGS), number of children (NCh) and educational attainment (EA) measured as the number of years of education. Traits were adjusted for age at recruitment, sex, recruitment centre, year of birth, genotyping batch, socioeconomic status measured by the Townsend deprivation index and population structure measured by 10 genetic principal components estimated from HM3 SNPs. Inbreeding depression was estimated within unrelated EI controls using two inbreeding measures: *F*_UNI_ and *F*_ROH_. Resulting estimates were used to linearly predict the reduction in EI cases. Vertical bars around predictions corresponds to 99.5% confidence interval as the significance was defined here at *p* < 0.05/10

We then assessed whether the observed reduction in these ten traits was consistent with inbreeding depression quantified within EI controls. Under the assumption that inbreeding depression results only from directional dominance effects of deleterious alleles or heterozygote advantage (overdominance), phenotypes are expected to decline linearly with increased inbreeding. However, if epistasis contributes to inbreeding depression^38,39^ or if causal variants for inbreeding depression are rarer^1^, a nonlinear relationship could be observed in particular for large inbreeding coefficients. To test this hypothesis we first estimated inbreeding depression in 345,276 EI controls unrelated with each other and unrelated with the 125 EI cases. For each of the 10 control traits, we then compared the phenotypic mean in the 125 EI cases, with a linear prediction based on the estimate of inbreeding depression in EI controls. For this analysis inbreeding depression was also estimated using an alternative inbreeding measure (*F*_UNI_), which we previously showed to be more powerful for detecting inbreeding depression^4^. The latter analysis did not reveal a significant deviation from the linear prediction (Wald-test *p* > 0.005) regardless of the inbreeding measure used, which therefore underlines that the observed phenotypic reduction in EI cases is consistent with inbreeding depression observed within EI controls (Fig. 4). This also suggests that causal variants contributing to inbreeding depression in those traits are likely well-tagged (i.e., correlated) by common variants in the population. However, we acknowledge that the estimate of inbreeding depression from the EI cases present in the UKB might be too low if, as seems plausible, they are a relatively healthy sample from the population of all EI cases in the UK^28^.

We next analysed the number of diseases diagnosed in an individual as an overall measure of health (Methods). We used overdispersed Poisson regression to estimate the relative risk (RR) of being diagnosed with at least one disease in EI cases as compared to EI controls. We found a RR of ~1.44 (Wald-test *p* = 3.6 × 10^−5^; Table 3). To minimise potential biases due to partial or differential disease reporting between UKB participants, we re-estimated RR in individuals with at least one disease diagnosed. This analysis included only 110 of the 125 EI cases identified and similarly showed a reduced but still significant RR ~1.34 (Wald-test *p* = 4.4 × 10^−4^; Table 3). In summary, we confirm that EI produces offspring with reduced stature (height), cognitive function (EA, FIS, and MTCIM), AA, muscular fitness (HGS), and lung function (PEF), consistent with a linear decline in these traits as inbreeding increases. We also provide additional evidence that offspring resulting from EI have increased risk for developing any type of disease.

### Social context of EI cases

We tested the association between EI and the Townsend depression index, which quantifies the level of socioeconomic deprivation in areas where UKB participants live. We found significant evidence that EI is enriched in more socioeconomically deprived area (odds ratio: 1.22; CI_95%_: [1.16–1.29]; Wald-test *p* = 2.6 × 10^−13^), consistent with a previous study^13^, which reported association between *F*_ROH_ and the same index in the UKB.

We further investigated the social contexts in which EI arose. For that we compared different characteristics of the parents of EI cases with that of the parents of EI controls. We found that 14.5% (i.e., 18/124, 1 missing value) of EI cases vs. 1.5% of controls reported to be adopted as a child (Fisher exact test *p* = 7.3 × 10^−13^). Given the significance of this difference we therefore focused all subsequent comparisons in nonadopted participants (106 EI cases vs. 339,241 EI controls) in order to minimise biases due to differential reporting of parental traits.

Previous studies^40^ have suggested that low EA of parents could be a cause of inbreeding in the population. Given that EA of parents of UKB participants has not been measured, we therefore tested this hypothesis by comparing mean genetic predictors of EA in UKB participants between EI cases and EI controls. Note that mean genetic predictor of EA is an estimate of the parental average for this trait. We found no statistical evidence that the mean genetic predictor of EA in EI cases deviate from that of EI controls (*t* test *p* = 0.538; Table 3). In fact, the mean genetic predictor of EA in EI cases approximately equals the median of the EA genetic predictor distribution in EI controls, which highlights that EI cases are not outliers on this scale. Besides EA, we then used overdispersed Poisson regression to compare the number of diseases (Online method) reported in parents of EI cases vs. parents of controls, which we used as another proxy for socioeconomic status of parents. We found no significant evidence that parents of EI cases are enriched for comorbidities as compared to parents of EI controls (RR ~0.96; Wald-test *p* = 0.507; Table 3). However, this observation must be interpreted with caution as it may simply reflect that EI cases observed in the UKB may be from more healthier background as compared to EI in the general population. Although additional information on parents of UKB participants was available (i.e., age of parents or age when parent died), missing values rates were often too large (>50%) among EI cases to draw reliable inference. Finally, we investigated if EI cases were geographically clustered, but found no significant association between EI and birth location (North coordinate: Wald-test *p* = 0.15; East-coordinate: Wald-test *p* = 0.08). Note that the absence of geographical clustering that we report only applies to these extreme events and could also reflect lack of statistical power as we still observed variance in mean *F*_ROH_ between different geographical areas of the UK. Altogether, although we observed that EI is more prevalent in more socially deprived areas of the UK, our results point to an absence of evidence that social and geographical stratification of parents contribute to the prevalence of EI in the population.

## Discussion

In this study, we estimated a prevalence of EI of ~1/3652 in individuals of European ancestry born in the UK between 1938 and 1967. Importantly, our estimate of the UK prevalence of EI is likely downwardly biased partly because of the ascertainment of UKB participants, who are on average healthier and more educated than the rest of the UK population^28^. It also worth mentioning that our estimate only accounts for mating between close relatives that have led to viable offspring. Altogether, our findings suggest that the prevalence of EI in the population is small and that very large observational studies are required to quantify it accurately.

We aimed in this study to quantify EI as it can routinely be detected in clinical screenings if genotypes are available. Therefore, we followed guidelines from the American College of Medical Genetics and Genomics, which recommend the use of both *F*_ROH_ and a threshold at 0.1. Nevertheless, we acknowledge that ACMG guidelines may be suboptimal with respect to detection of EI and that other approaches could have been in implemented^41,42^. We found in our simulations that a threshold 0.1 may in fact be too conservative, while using a threshold of ~0.08 is optimal with respect to specificity and sensitivity to detect EI (Fig. 1).

In addition, our study has addressed theoretical questions regarding the distribution of genomic segments homozygous-by-descent, which are classically approximated using long ROHs. Indeed, we explored how the distribution of long ROHs can be utilised to infer mating types underlying EI. Although we only applied threshold-based methods, we found that such simple approaches perform quite well in our simulations (AUC > 0.95). However, it is worth mentioning that previous studies have addressed a similar question using more elaborate models. For example, Druet and Gautier^42^ introduced a model-based approach which assumes individual genomes to be a mosaic of HBD and non-HBD segments, and allows HBD segments to originate from different ancestors at different time points. The aim of their method is therefore to estimate simultanesouly the age and the HBD status of genomic segments. Note that knowing the age of an HBD segments directly informs the likelihood of certain mating types.

One similarity between Druet and Gautier’s approach and ours, is that we both assumed the distribution of HBD segments to follow a mixture of exponential distributions. However, our approach relies on observed ROHs, which we have assumed to be HBD, whereas Druet and Gautier models HBD segments as unobserved states of a hidden Markov chain. Consequently, their inference is likely more robust to biases from ROHs calling, which often requires arbitrary choices to be made (e.g., minimum length of ROHs, minimum distance between ROHs and number of occasional heterozygotes allowed). On the other hand, the Druet and Gautier method relies on the assumption that the length of HBD segments follows an exponential distribution as a consequence of assuming a constant recombination rate. Our study provides a simulation-based (using observed genetic maps) and an empirical quantification of the length distribution of long genomic segments identical-by-descent, which we found to best fit a mixture of two exponential distributions. Therefore, our results confirm that the assumption of constant recombination rate is inappropriate for describing segments length distribution^33^, and we show that mixtures of exponential distributions provide a mathematically tractable framework to accommodate arbitrary recombination maps. We note that Druet and Gautier acknowledged that violation of the assumption of a constant recombination rate across the genome could limit the interpretation of their model parameters.

We showed in this study that the reduction in measured values of multiple complex fitness-related traits resulting from EI is consistent with inbreeding depression estimated within EI controls, who still harbour ROHs in their genome^43^. If inbreeding depression in EI controls is well estimated then the latter finding would suggest that gene × gene or gene × environment interactions contribute little to inbreeding depression in the traits analysed and also that variants causal of inbreeding depression in these traits are well tagged (i.e., correlated) by common SNPs. However, because of ascertainment of UKB participants who are on average healthier and more educated than the general population^28^, estimates of inbreeding depression in UKB participants may also be underestimated. Moreover, Curik et al.^44^ showed using computer simulations that the absence or presence of a nonlinear relationship between inbreeding and traits should be interpreted with caution in particular when inbreeding depression is estimated using an inbreeding measure which only partially reflects realised autozygosity, as is the case for *F*_ROH_.

Lastly, we attempted to quantify the contribution of social contexts to the prevalence of EI. Despite the sparsity of parental information for EI cases, we found no evidence that EI is more prevalent in health-deprived families nor that low education contributes to increase the likelihood of EI in the population. In conclusion, our study provides an objective quantification of EI in the UK population and shed lights on its causes and phenotypic consequences.

## Methods

### SNP genotyping

We used genotyped and imputed allele counts at 16,652,994 SNPs imputed to the Haplotype Reference Consortium^45^ imputation reference panel, in 487,409 participants of the UKB^29,46^. Extensive description of data can be found here^26^. We restricted our analysis to 456,414 participants of European ancestry identified using projected principal components based on sequenced participants of the 1000 genomes projects with known ancestry^26^. This subset of the UKB contains 348,502 conventionally unrelated participants, i.e., whose estimated pairwise SNP-based GRM < 0.05, estimated using 1,124,803 common (minor allele frequency (MAF) ≥ 1%) HapMap3^47^ SNPs using GCTA (v1.9)^48^. The North West Multi-Centre Research Ethics Committee (MREC) approved the study and all participants in the UKB study analysed here provided written informed consent.

### Polgenic predictor of EA

We used estimated SNP effects from the Lee et al.^49^ GWAS of EA to calculate polygenic score predicting EA. HM3 SNP effects were re-estimated after excluding data from the UKB. Marginal SNP effects were then transformed into conditional SNP effects using the LD-pred method^50^ assuming all SNPs to be causal. The latter analysis used genotypes at HM3 imputed SNPs of ~300,000 unrelated UKB participants as linkage disequilibrium reference panel.

### ROH detection

ROH were called using only 301,412 SNPs genotyped in 456,414 UKB participants of European descent. These SNPs were filtered on missingness rate (missingness < 1%), MAF > 5% and Hardy–Weinberg equilibrium test *p* value > 0.0001. As in previous studies^4,8,10^, we used the following PLINK (versions 1.07 and 1.9)^51,52^ command to call ROH: --maf 0.05 --homozyg --homozyg-density 50 --homozyg-gap 1000 --homozyg-kb 1500 --homozyg-snp 50 --homozyg-window-het 1 --homozyg-window-missing 5 --homozyg-window-snp 50. That command detects ROHs at least 1.5 Mb long, at least 1 Mb apart from one another, containing at least 50 SNPs, and such that SNPs overlapping ROH can have at most 5 missing values and 1 occasional heterozygote. Once ROHs detected, we calculate an inbreeding measure *F*_ROH_ for each individual by dividing the cumulated length of ROH in Mb by an estimate of the length of the human autosome, i.e., ~2881 Mb under genome build hg19. Note that this estimate of autosome length may vary between genome builds, and therefore may impact the number of individuals detected above a given threshold.

### Simulation of EI

To simulate EI we used 972 independent (GRM < 0.05) trios (both parents and one offspring) out of 1066 identified in the UKB^29^. We used the same set of 301,412 genotyped and quality-controlled SNPs as to call ROH to phase haplotypes using SHAPEIT 2 with the following options: --duohmm -W 5 -T 10 and using genetic maps from the 1000 Genomes (1KG) Project phase 3 (hg19, see URL)^23^. We considered eight different mating types (pedigrees): mating between unrelated individuals (i.e., any pair among the unrelated 972 samples), between first-cousins, between double-first cousins, between grandchildren and grandparents, between uncles/aunts and nieces/nephews, between HSs, between fullsibs and between parents and offspring. Nonetheless, we describe here the case of PO mating. First, we sample a random pair of individuals (denoted P_1_ and P_2_) out of 972 × 971/2 = 471,906 possible pairs. We then create recombined chromosomes from haplotypes of P_1_ and P_2_. For all genetic intervals defined in the 1KG genetic maps, we sample the Bernoulli distributed indicator of the presence of a recombination breakpoint with probability equal to 0.01 × genetic distance of the interval in Morgan(s). Once the recombined chromosomes of the offspring O of P_1_ and P_2_ are simulated, we then repeat this procedure to simulate an offspring resulting from mating of O with one of the parent, i.e., P_1_ or P_2_. To then mimic real data, which contain genotyping errors, we also add a random number of errors to the simulated genotypes. The number of errors is sampled from a Poisson distribution with a mean corresponding to the mean number of genotyping errors estimated, for each chromosome, from comparing genotypes of 168 twin pairs (Supplementary Table 2). We found overall a genotyping error at quality controlled SNPs ~4.5 × 10^−4^, which is orders of magnitude larger than the rate of new somatic mutation, which was previously estimated around ~2.8 × 10^−7^ in human fibroblasts^53^. Therefore, somatic mutation would have a negligible effect on ROH calling given the set of parameters that we used.

### Association with phenotypes measured in the UKB

We used GCTA with the --ibc command to estimate for each UKB participants the correlation between uniting gametes^48^. That statistic denoted *F*_UNI_ (also known as “Fhat3”) is an estimate of inbreeding using allele frequencies in the current population and was previously shown to be more powerful to detect ID^4^. We nonetheless condidered *F*_ROH_ as a reference inbreeding measure in this study in accordance with the ACMG guidelines. We tested the association between inbreeding measures (*F*_ROH_ and *F*_UNI_) and traits using linear regression adjusted for age at recruitment (UKB field 21022–0.0), sex, assessment centre (UKB field 54–0.0), genotyping chip and batch, year of birth (UKB field 34–0.0), socioeconomical status measured by the Townsend deprivation index (UKB field 189–0.0) and 10 genetic principal components calculated using PLINK 2.0. Analyses were performed in 345,276 unrelated EI controls (*F*_ROH_ < 0.01). Traits were pre-adjusted and inverse normal transformed and phenotypic values larger than >4 standard deviations were excluded. UKB identifiers for tested traits are: height (UKB field 50-0.0), hip-to-waist ratio (HWR: ratio of UKB field 49-0.0 over UKB field 48-0.0), HGS (average of UKB fields 46-0.0 and 47-0.0), lung function measured as the PEF (UKB field 3064-0.0), VA measured on log MAR scale (VA: average between UKB field 5201-0.0 and UKB field 5208-0.0), auditory acuity measured as te speech reception threshold (AA: average between UKB field 20,019-0.0 and UKB field 20,021-0.0), number of years of education (EA), fluid intelligence score (FIS: UKB field 20,016-0.0), cognitive function measured as the mean time to correctly identify matches (MTCIM: UKB field 20,023-0.0) and fertility measured as the number of children (NCh: for males UKB field 2405-0.0 and for females UKB field 2734-0.0). To test the association between number of diseases diagnosed and inbreeding, we used overdispersed Poisson regression implemented in R 3.2.0 (*glm* function with option family = “quasipoisson”). Number of diseases diagnosed was estimated as the number International Classification of Diseases, Tenth Revision (ICD10) codes reported for UKB participants. We also analysed reported illnesses in fathers and mothers of UKB participants (UKB fields 20,107 and 20,110, respectively) as measure of health deprivation in the family. Illnesses of parents were reported among 12 groups of diseases (URLs). We created for each participant a count of diseases in both parents. Analysis were adjusted for adoption status (UKB field 1767) and missing values on the parental diseases were excluded.

### Length distribution of ROHs

We estimated the length distribution of ROHs using a mixture of exponential distributions with a number of components from 1 to 10. Given that only ROHs larger than 1.5 Mb are detected, we therefore analysed lengths of ROHs in Mb minus the minimum threshold (as in Fig. 2). This choice is justified by following property of exponential distributions. If *X* follows an exponential distribution of rate *λ*, then *Y* = *X*|X > *s*, i.e., the truncated distribution of *X* with values larger than a given threshold *s*, is such that (Y-*s*) also follows an exponential distribution with the same rate (*λ*) as *X*. Estimation of mixture distribution was performed using the R package *Renext*. Model selection was performed using BIC criterion.

### Discriminate PO vs. FS mating from ROH length distribution

Given a collection of autosomal ROH segments lengths, we developed a method for estimating the proportion *π*_*PO*/*FS*_ of these segments resulting from PO vs. FS mating. We denote *f*_*PO*_ and *f*_*FS*_ as the probability density functions of (ROHs) segments length under PO and FS, respectively. We assume that the length distribution of the set of ROHs used for inference is a mixture of *f*_*PO*_ and *f*_*FS*_ and we denote *π*_*PO*/*FS*_ as the mixture proportion. We also assumed *f*_*PO*_ and *f*_*FS*_ to be known so that the parameter of interest, i.e., that we want to estimate, is *π*_*PO*/*FS*_.

The log-likelihood *l*(*x*;*π*_*PO*/*FS*_) of one segment of length *x* can be written as

1 $${l\left( {x;\pi _{PO/FS}} \right) = {\mathrm{log}}\left[ {\pi _{PO/FS}f_{PO}(x) + \left( {1 - \pi _{PO/FS}} \right)f_{FS}(x)} \right] = {\mathrm{log}}\left[ {\pi _{PO/FS}\left( {f_{PO}(x) - f_{FS}(x)} \right) + f_{FS}(x)} \right]}.$$

From that we can write the Fisher information as

2 $${\Bbb E}\left[ { - \frac{{\partial ^2l\left( {X;\pi _{PO/FS}} \right)}}{{\partial \pi _{PO/FS^2}}}} \right] = {\Bbb E}\left[ {\left( {\frac{{f_{PO}(X) - f_{FS}(X)}}{{\pi _{PO/FS}\left( {f_{PO}(X) - f_{FS}(X)} \right) + f_{FS}(X)}}} \right)^2} \right],$$

where *X* ’s probability log density function is *l*(*x*;*π*_*PO*/*FS*_). Therefore the asymptotic variance of the maximum likelihood estimator $\hat \pi _{PO/FS}$ of *π*_*PO*/*FS*_ would be

3 $${\mathrm{var}}[\hat \pi _{PO/FS}] \approx \frac{1}{N} \times \left\{ {{\Bbb E}\left[ {\left( {\frac{{f_{PO}(X) - f_{FS}(X)}}{{\pi _{PO/FS}\left( {f_{PO}(X) - f_{FS}(X)} \right) + f_{FS}(X)}}} \right)^2} \right]} \right\}^{ - 1}.$$

where *N* is the number of segments used to estimate *π*_*PO*/*FS*_.

We use parameters from Table 2 to characterise *f*_*PO*_ and *f*_*FS*_. Each of these two distributions were approximated using mixtures of two exponential distributions which parameters were estimated from >648,125 simulated ROHs under PO and FS. Conditional on *f*_*PO*_ and *f*_*FS*_, estimating *π*_*PO*/*FS*_ is therefore a straightforward univariate optimisation problem. We used Eq. (3) to quantify the standard error of $\hat \pi _{PO/FS}$. The expectation in Eq. (3) was approximated using one million Monte Carlo simulations conditional on $\hat \pi _{PO/FS}$, *f*_*PO*_ and *f*_*FS*_.

### URLs

For Crime Survey for England and Wales, see https://www.ons.gov.uk/file?uri=/peoplepopulationandcommunity/crimeandjustice/datasets/sexualoffencesappendixtables/yearendingmarch2017/sexualoffencesappendixtablesmarch2017.xls

(Table 8; Offence code 23, total number of cases is 11,196).

For Population sizes in England and Wales, see

https://www.ons.gov.uk/peoplepopulationandcommunity/populationandmigration/populationestimates.

For Educational attainment in the UK from 2011 Census, see

http://www.nomisweb.co.uk/census/2011/DC5102EW/view/2092957703?rows=c_age&cols=c_hlqpuk11.

For Genetic maps from the 1,000 Genomes Project, see ftp://ngs.sanger.ac.uk/production/samtools/genetic-map.tgz.

For UK Biobank groups of diseases affecting parents, see

https://biobank.ctsu.ox.ac.uk/crystal/coding.cgi?id=1010.

### Reporting summary

Further information on research design is available in the Nature Research Reporting Summary linked to this article.

## Supplementary information

Supplementary Information

Reporting Summary

## Data Availability

This study makes use of genotype and phenotype data from the UK Biobank data under project 12505. UKB data can be accessed upon request once a research project has been submitted and approved by the UKB committee. We also provide data source for generating all figures.

## Code availability

We provide the R script that we used to simulate genotypes of EI cases individuals from a collection of phased haplotypes in SHAPEIT2 format at https://github.com/loic-yengo/InbreedingSimulationR. We also provide R scripts for generating all figures.
