# Supplementary material for: Extreme inbreeding in a European ancestry sample from the contemporary UK population
Source: Nat Commun. 2019 Sep 3;10:3719. doi: 10.1038/s41467-019-11724-6 (PMC6722066; doi:10.1038/s41467-019-11724-6)
Supplement: Supplementary file 1 — Supplementary Information [file 41467_2019_11724_MOESM1_ESM.pdf]

Supplementary Tables

Inbreeding measures	Number of EI cases (F>0.1)	Prevalence of EI (95% CI)
F_{ROH}		
ROH>1.5 Mb		
autosome only	125	1/3652 (1/4428 - 1/3106)
autosome + X-chromosome in females	131	1/3485 (1/4204 - 1/2974)
ROH>2.0 Mb		
autosome only	115	1/3969 (1/4857 - 1/3355)
autosome + X-chromosome in females	119	1/3836 (1/4676 - 1/3251)
ROH>5.0 Mb		
autosome only	98	1/4658 (1/5807 - 1/3887)
autosome + X-chromosome in females	100	1/4565 (1/5677 - 1/3816)
F_{UNI}		
MAF>0.001	122	1/3742 (1/4549 - 1/3177)
MAF> 0.01	123	1/3711 (1/4508 - 1/3153)
MAF> 0.05	127	1/3594 (1/4351 - 1/3061)
F_{HOM}		
MAF>0.001	134	1/3407 (1/4101 - 1/2912)
MAF> 0.01	134	1/3407 (1/4101 - 1/2912)
MAF> 0.05	130	1/3511 (1/4240 - 1/2995)

Supplementary Table 1. Estimated prevalence of extreme inbreeding (EI) using different SNP-based inbreeding measures. For each inbreeding measure F, EI is called when F>0.1. Three measures of inbreeding are considered: F_{ROH} measures the percentage of one's genome under runs of homozygosity (ROH); F_{UNI}, which measures the correlation between uniting gametes and F_{HOM}, which measures the excess of homozygosity under Hardy-Weinberg equilibrium. F_{ROH} was calculated using ROHs of different lengths: >1.5 Mb, >2 Mb and >5Mb. All ROHs were called using 301,412 quality-controlled genotyped autosomal SNPs (and 8,579 X-chromosome SNPs) with minor allele frequency (MAF) >5%. F_{UNI} and F_{HOM} were calculated using three nested sets of quality-controlled genotyped autosomal SNPs: 538,010 SNPs with MAF>0.1%, 500,082 SNPs with MAF>1% and 301,412 SNPs with MAF>5%. All approaches yield consistent estimate of a prevalence of EI in of ~1/3500.

Chromosome	Number of SNPs	Mean number of errors	Standard Deviation	Per SNP Error Rate ($\times 10^{-4}$)
1	23,690	20.5	19.6	4.3
2	23,461	22.0	21.4	4.7
3	19,914	19.2	18.5	4.8
4	18,442	19.3	18.3	5.2
5	17,670	17.4	16.6	4.9
6	21,846	18.8	18.5	4.3
7	16,467	15.3	14.8	4.7
8	15,169	14.7	14.0	4.8
9	13,436	11.9	11.2	4.4
10	14,811	12.6	12.5	4.3
11	14,945	12.4	12.1	4.2
12	14,454	13.2	12.5	4.6
13	10,234	10.1	9.7	4.9
14	9,674	8.7	8.8	4.5
15	9,717	8.4	8.3	4.3
16	10,975	8.9	9.2	4.0
17	10,368	7.7	8.1	3.7
18	9,137	8.9	8.9	4.9
19	8,839	6.4	6.8	3.6
20	8,161	6.2	6.5	3.8
21	4,679	4.1	4.2	4.4
22	5,323	3.6	4.0	3.4
Autosome	301,412	270.3	254.1	4.5

Supplementary Table 2. Distribution of genotyping errors per chromosome from the comparison of genotypes of 178 monozygotic twin pairs among UK Biobank participants. Reported statistics are the mean and standard deviation over 178 twin pairs of the number of discordances, and per SNP error rates, which is defined as the mean number of mismatches between twins (e.g. genotyping errors or somatic mutations) divided by twice the number of SNPs. Error rates were calculated over a set of 301,412 quality controlled SNPs as described in the Method section.

Supplementary Figures

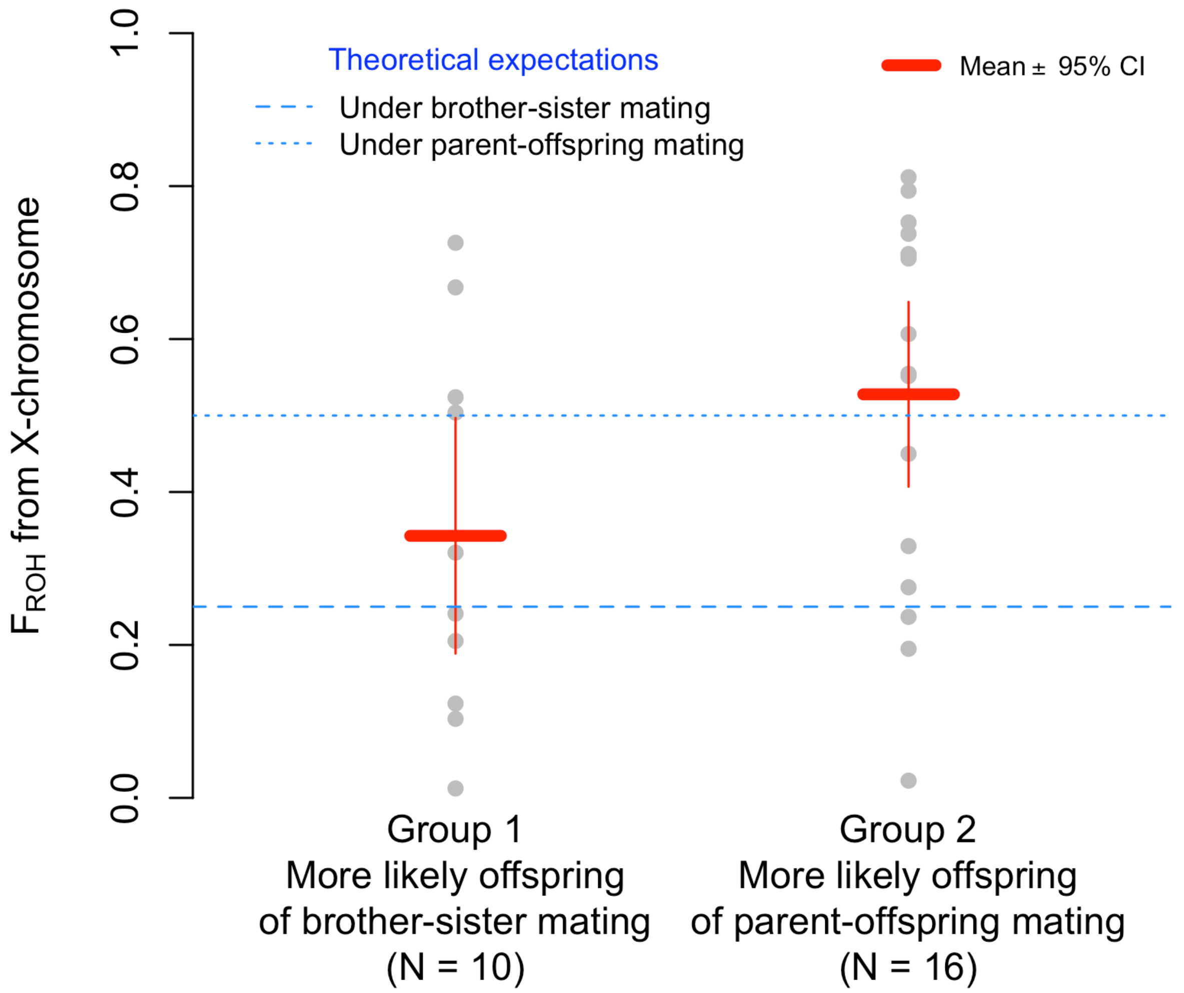

Supplementary Figure 1. Distribution of inbreeding coefficient (F_{ROH-X}) estimated from runs of homozygosity (ROH) observed on X-chromosomes of 26 female extreme inbreeding (EI) cases. F_{ROH-X} is defined as the cumulated length (in Mb) of ROHs, divided the expected length of the X-chromosome, here ~151 Mb. Female EI cases were stratified into two groups (Group 1 and Group 2) depending on whether the likelihood of their autosomal ROH segments lengths is larger under parent-offspring as compared to under brother-sister mating. Group 1 contains N=10 EI cases which distribution of autosomal ROH segments lengths best fits the estimated length distribution of ROH under FS mating. The mean F_{ROH-X} in Group 1 is 0.34 ($CI_{95\%}$:[0.19; 0.49]). Group 2 contains N=16 EI cases and the mean F_{ROH-X} is 0.53 ($CI_{95\%}$:[0.41; 0.65]). Theoretical expectations of F_{ROH-X} under FS (E[F_{ROH-X}] = 0.25) and PO (E[F_{ROH-X}] = 0.5) mating are represented by dotted lines.

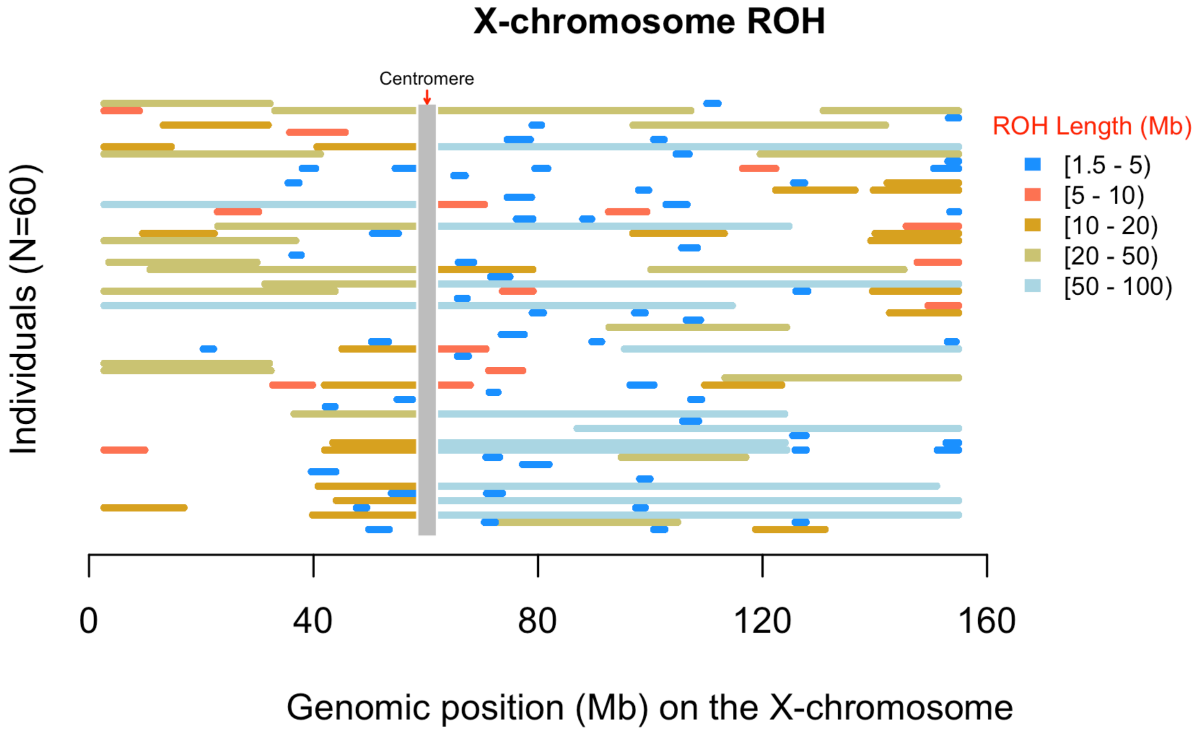

Supplementary Figure 2. Genomic position of runs of homozygosity (ROHs) detected on X-chromosomes of 60 female EI cases (autosomal F_{ROH}>0.1). Each row, with possibly multiple segments, represents a unique participant. ROHs are grouped in 5 length categories (between 1.5 Mb and 5 Mb, between 5 Mb and 10 Mb, between 10 Mb and 20 Mb, between 20 Mb and 50 Mb and between 50 Mb and 100 Mb) and coloured accordingly.
